# Pulse Amplitude Measured with a Portable Laser Doppler Flowmeter Is Useful for Screening of Dialysis Patients for Peripheral Arterial Disease: An Observational Study

**DOI:** 10.3400/avd.oa.21-00126

**Published:** 2022-12-25

**Authors:** Makoto Saito, Hiroomi Jingu, Hidefumi Osawa, Yusuke Oyama, Toshiyuki Tanaka, Akihiko Shiono, Masami Machida

**Affiliations:** 1 Department of Clinical Engineering, Public Tomioka General Hospital, Tomioka, Gunma, Japan; 2 Department of Urology, Public Tomioka General Hospital, Tomioka, Gunma, Japan

**Keywords:** peripheral arterial disease, laser Doppler flowmeter, pulse amplitude

## Abstract

**Objective:** The objective of this study was to use a portable laser Doppler flowmeter (LDF) to measure the toe blood flow and pulse amplitude as a screening test for peripheral arterial disease (PAD) in dialysis patients and compare the diagnostic abilities of the aforementioned parameters measured using an LDF with those of the ankle-brachial index (ABI) and toe brachial index (TBI).

**Methods:** The 14 patients in this retrospective study received maintenance hemodialysis (HD). We measured the blood flow and pulse amplitude on the ventral side of the first toe with a portable LDF while the patients were undergoing an HD session. The correlations between the blood flow/pulse amplitude in the toe and the ABI/TBI were examined.

**Results:** Both the ABI and TBI had a strong correlation with pulse amplitude. The sensitivity and specificity of the pulse amplitude measured with the LDF for detecting PAD in HD patients as determined by a receiver operating characteristic curve analysis were 1.00 and 0.88, respectively.

**Conclusion:** Measuring the pulse amplitude in the toe with a portable LDF may serve as a simple and useful screening test for PAD in HD patients.

## Introduction

In general, measurement of the ankle-brachial index (ABI) is used as the screening test for the peripheral arterial disease (PAD).^1)^ An angiographic study conducted in nonhemodialysis (HD) patients using the diagnostic criterion for PAD of ABI<0.9^2)^ showed extremely high sensitivity and specificity of ABI for the diagnosis of PAD of 95% and 100%, respectively. However, a multidetector-row computed tomography (CT) study conducted in HD patients showed that the sensitivity of ABI for the detection of PAD in HD patients was very low (29.9%), even though the specificity was still 100%.^3)^ Diabetic and HD patients often show high levels of calcification of the tunica media in the central arteries, below the knee vessels, which often leads to falsely normal levels or sometimes abnormally high values of the ABI of 1.4 or higher.^3)^ Thus, it may be difficult to expect a high diagnostic ability of ABI for PAD in HD patients.

Since calcification of the tunica media in the arteries rarely extends to the toe arteries, the toe brachial index (TBI) has been used as an index to evaluate impaired blood circulation in the peripheral arteries, and its usefulness has been reported in both diabetic patients^4)^ and HD patients.^5,6)^ Some drawbacks of the measurement of the TBI are that it cannot be measured in the presence of involuntary movements, small-muscle cramps, arrhythmias, or blood pressure ≤40 mmHg. Recently, other screening tests have been attempted to determine skin microcirculation adequacies, such as skin perfusion pressure and transcutaneous oxygen tension. However, the procedures for measuring these indices are complicated, so they are not widely used. None of these tests were performed during dialysis sessions.

There have been some reports in recent years suggesting the usefulness of monitoring the blood flow in the lower limbs using a portable laser Doppler flowmeter (LDF), which can be easily used to evaluate the skin microcirculation.^7–9)^ Evaluation using a portable LDF can be easily performed, even during a dialysis session. The present study aimed to clarify the usefulness of measuring the toe blood flow and pulse amplitude with a portable LDF as a screening test for PAD in dialysis patients compared to the diagnostic abilities of ABI and TBI.

## Subjects and Methods

### Subjects

**Figure 1:**
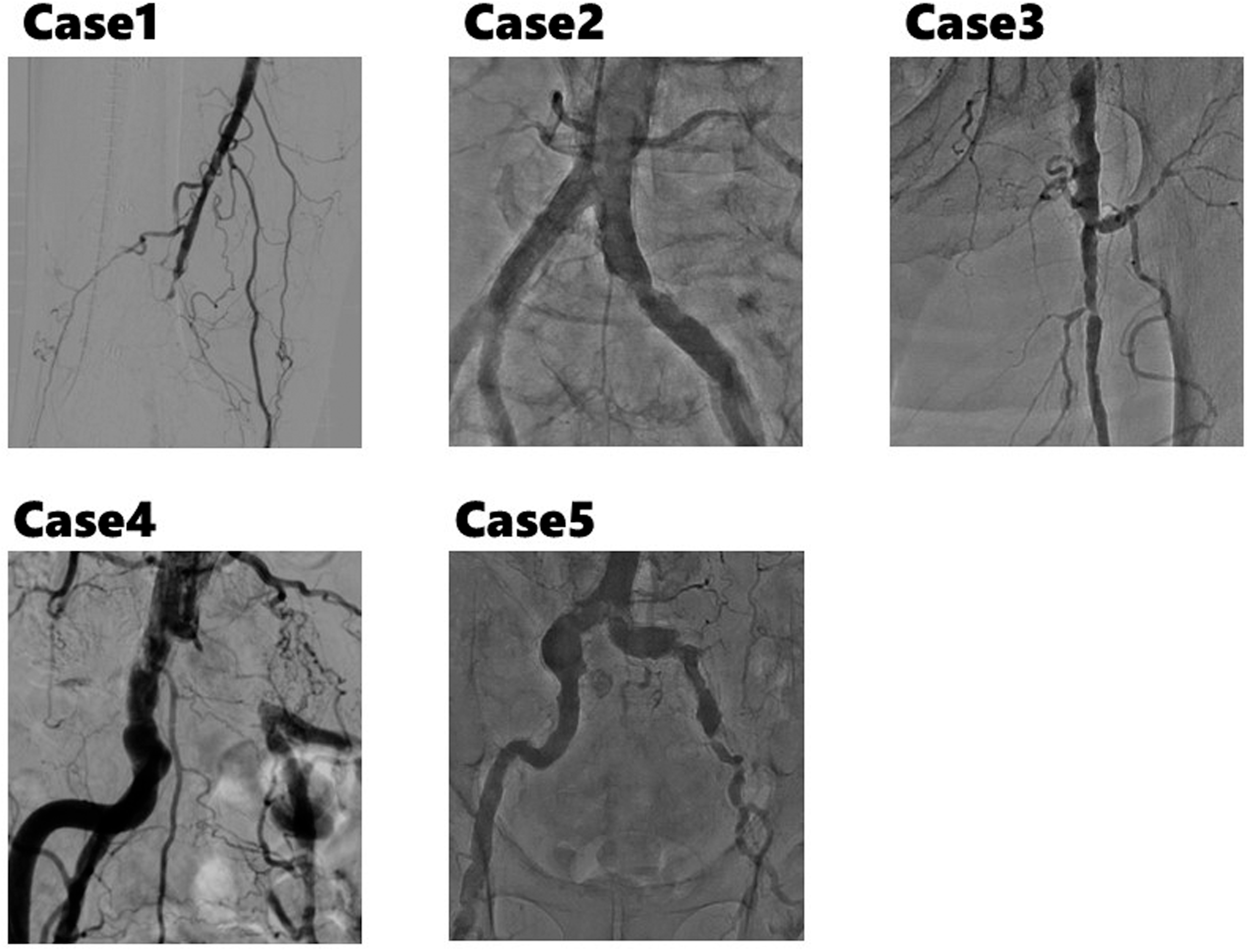
Fig. 1 Angiographic images of 5 hemodialysis patients with peripheral arterial disease. Case 1: Occlusion of the right popliteal artery; Case 2: 75% stenosis of the left common iliac artery; Case 3: 75% stenosis of the left common iliac artery; Case 4: Occlusion of the left common iliac artery; Case 5: Occlusion of the left superficial femoral artery.

A total of 14 patients on maintenance HD were enrolled in this study. The mean age was 65.2 years, and the mean dialysis vintage was 9.1 years. The underlying kidney disease was chronic glomerulonephritis in seven patients, diabetic nephropathy in five patients, polycystic kidney disease in one patient, and nephrotic syndrome in one patient. The dialysis treatment method was predilution online hemodiafiltration in 11 patients and intermittent infusion hemodiafiltration in three patients. Among the subjects, five patients were diagnosed as having PAD based on the clinical symptoms in accordance with the Fontaine classification and the results of diagnostic angiography (**Fig. 1**).

### Protocol

The Medical Ethics Committee approved the study protocol at the Public Tomioka General Hospital (approval number: 2019-24). The study was conducted in compliance with the 1964 Helsinki Declaration and its later amendments or comparable ethical standards.

We measured the ABI and TBI before the start of an HD session and the blood flow and pulse amplitude on the ventral side of the first toe with a portable LDF (Pocket LDF, JMS Co., Ltd., Tokyo, Japan) during the HD session on the same day. The correlations between the blood flow/pulse amplitude in the toe and the ABI/TBI were investigated. Appropriate cutoff values for the sensitivity and specificity of the blood flow and pulse amplitude in the toe for PAD detection were determined by receiver operating characteristic (ROC) curve analysis.

### Measurement of the ABI and TBI

The ABI was measured in both legs before the dialysis session. The patients lay supine on a bed with blood pressure cuffs tied around their upper arm on the nonarteriovenous fistula side and ankles on both sides, and the blood pressure was measured using a BP-203RPEIII (FUKUDA COLIN Co., Ltd, Aichi, Japan). Next, a special cuff was fitted on the first toe on both sides, the toe systolic blood pressure was measured, and the TBI was calculated from the toe systolic blood pressure/brachial systolic blood pressure. Measurements were performed in the Blood Purification Room after the patients rested for 10 min at room temperature (25°C), avoiding eating and urinating.

### Measurement of the blood flow and pulse amplitude in the toe

**Figure 2:**
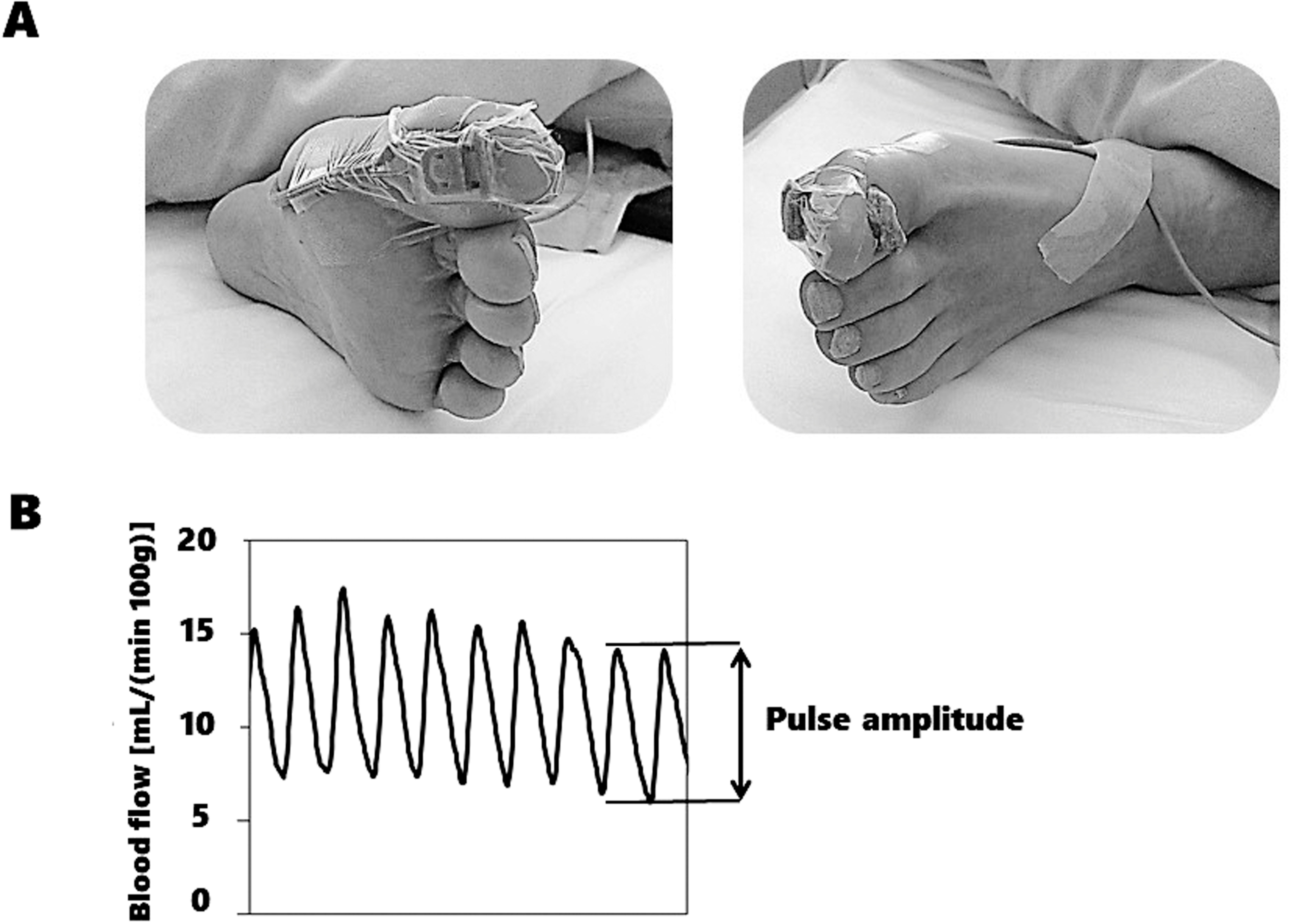
Fig. 2 Laser Doppler flow sensor and blood flow waveform. (**A**) Measurements were obtained using a portable laser Doppler flowmeter (LDF) probes attached to the first toe’s ventral side. (**B**) Example of a blood flow waveform recorded by the Pocket LDF.

We used a Pocket LDF to perform the measurements during the dialysis session after the ABI and TBI had been measured before the session. The measurements were obtained using LDF probes that were directly attached to the ventral side of the first toe (**Fig. 2**). LDF measurements are sensitive to temperature, but during dialysis, the measurements are made under constant room temperature (25°C) conditions, and the outside temperature exerts limited influence. The blood flow signals detected by a photodetector are transmitted to the main unit, where the measurement values are displayed. The flow rate was measured every 20 ms, the maximum and minimum peak values were detected, and the pulse amplitude was determined as the flow rate difference between the peaks. The obtained data were transferred via Bluetooth to dedicated application software (Pocket LDF Recorder). The average blood flow and pulse amplitude values for 4 h (during a dialysis session) were determined and used for the study. The results were expressed in mL/(min 100 g) for blood flow and pulse amplitude. Blood flow and pulse amplitude in the toe represent changes in the blood flow due to pulsations in the periphery, which may reflect the reactivity of the blood vessels up to the periphery. They may be useful for the detection of PAD.

### Statistical analysis

Statistical analyses were performed using Fisher’s exact test and Spearman’s rank correlation for multiple comparisons. P<0.05 was considered to be indicative of a significant difference.

## Results

First, to evaluate the correlation between the toe blood flow and pulse amplitude, the toe blood flow and pulse amplitude were measured using the LDF. There was no significant correlation between the blood flow and pulse amplitude (r=0.275; p=0.454, data not shown).

**Figure 3:**
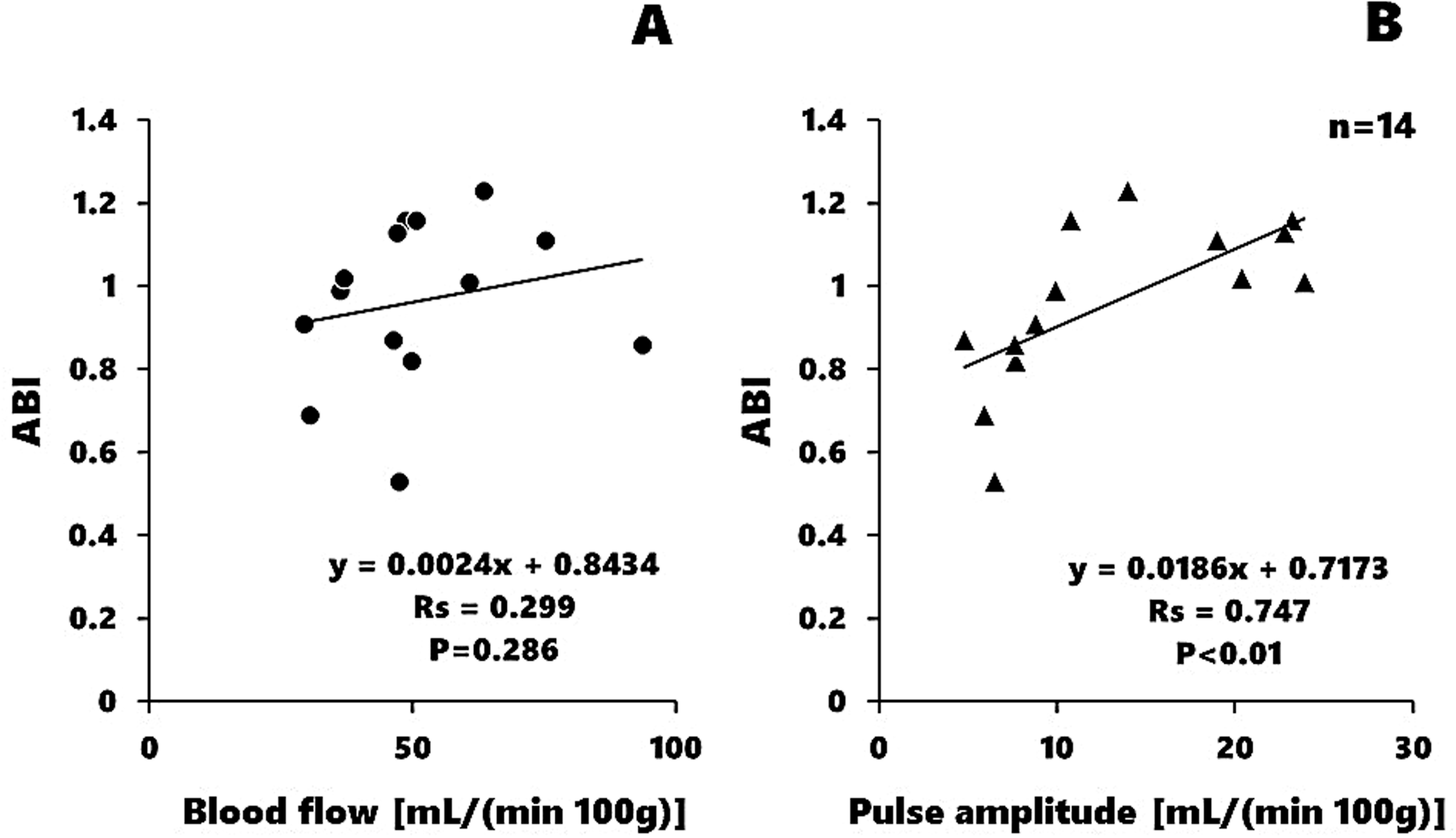
Fig. 3 Correlation between the blood flow (**A**)/pulse amplitude (**B**) and the ankle-brachial index (ABI). The blood flow was not correlated with the ABI (p=0.286), whereas the pulse amplitude showed a significant correlation with the ABI (p<0.01).

**Figure 4:**
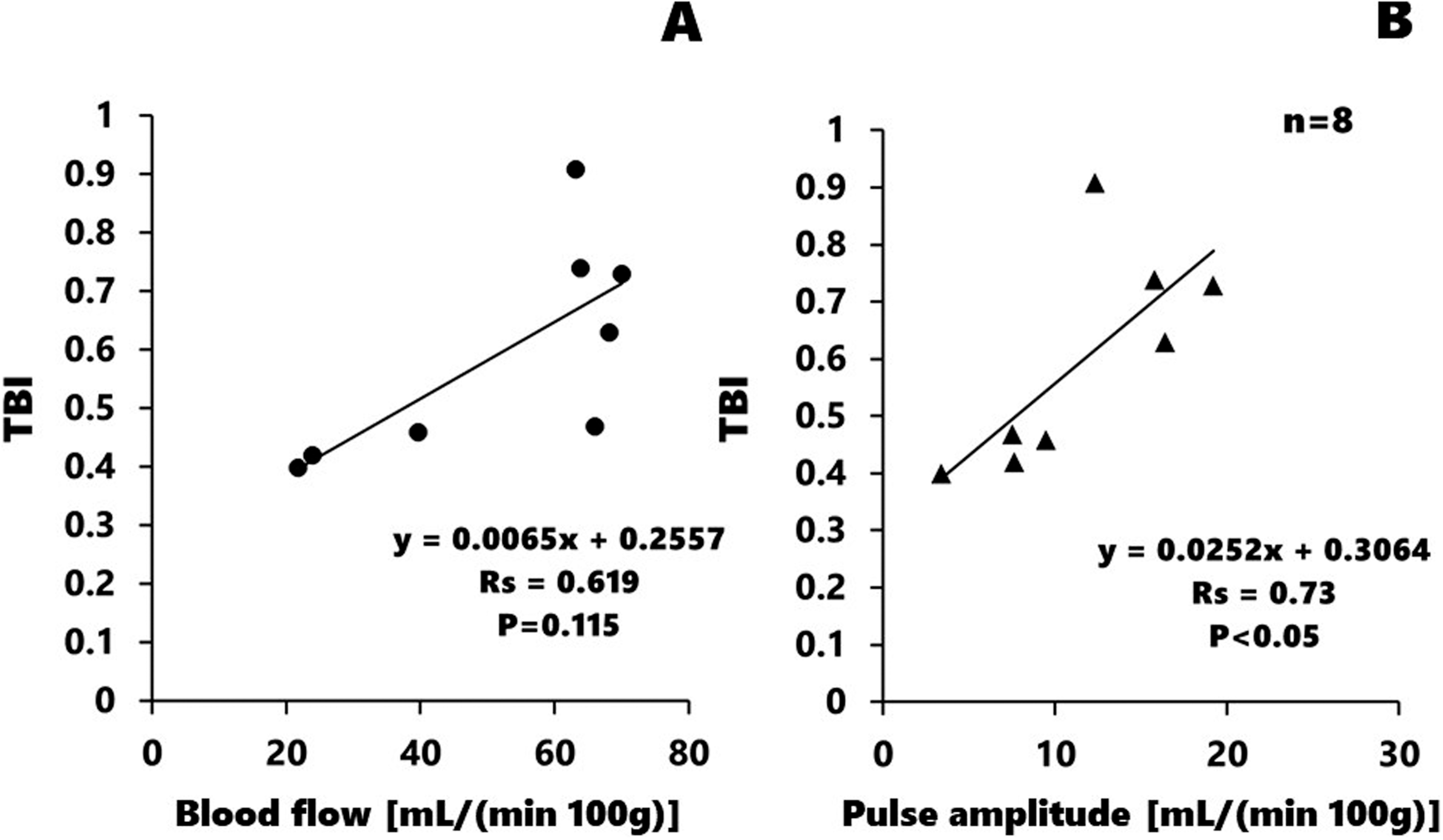
Fig. 4 Correlation between the blood flow (**A**)/pulse amplitude (**B**) and the toe brachial index (TBI). The blood flow was not correlated with the TBI (p=0.115), whereas the pulse amplitude showed a significant correlation with the TBI (p<0.05).

Then, the correlation between the ABI and toe blood flow/pulse amplitude was evaluated. No correlation was observed between the blood flow and ABI (r=0.299; p=0.286). However, the pulse amplitude showed a significant correlation with the ABI, decreasing in value as the ABI decreased (r=0.747; p<0.01) (**Fig. 3**). Then, the correlation between the TBI and toe blood flow/pulse amplitude was evaluated, which revealed the absence of any significant correlation between the TBI and toe blood flow (r=0.619; p=0.115), but a significant correlation between the pulse amplitude and the TBI (r=0.730; p<0.05); the pulse amplitude decreased as the TBI decreased (**Fig. 4**).

**Figure 5:**
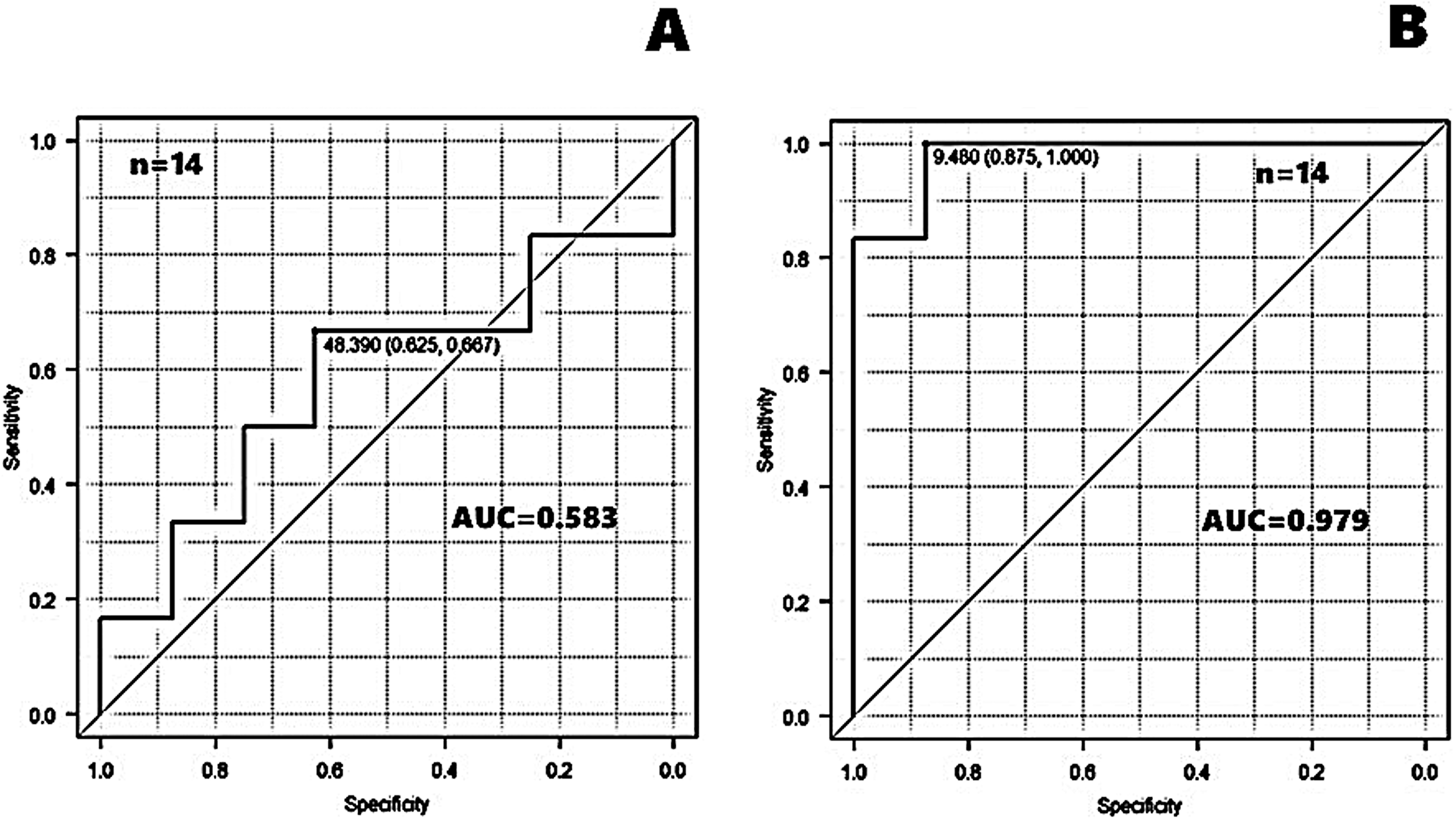
Fig. 5 Receiver operating characteristic curves of the blood flow (**A**) and pulse amplitude (**B**). The sensitivity and specificity of the blood flow measured in the toe to detect peripheral arterial disease (PAD) were 0.67 and 0.63, respectively. The sensitivity and specificity of the pulse amplitude measured in the toe to detect PAD were 1.00 and 0.88, respectively. The blood flow and pulse amplitude cutoff values were 48.4 mL/min and 9.5 mL/min, respectively.

A ROC curve analysis determined appropriate cutoff values for obtaining the maximum sensitivity and specificity of the toe blood flow and pulse amplitude for diagnosing PAD. We used the general reference cutoff values for ABI and TBI of 0.9 and 0.6, respectively. The sensitivity and specificity of ABI for the detection of PAD were 83% and 100%, respectively, and those of TBI were 100% and 75%, respectively. The sensitivity and specificity of the measured toe blood flow for diagnosing PAD were 67% and 63%, respectively, and those of the pulse amplitude were 100% and 88%, respectively. The ROC curve analysis indicated cutoff values for the blood flow and pulse amplitude of 48.4 mL/(min 100 g) and 9.5 mL/(min 100 g), respectively (**Fig. 5**). The pulse amplitude was 9.5 mL/(min 100 g) or lower in all the five patients diagnosed as having PAD. The average pulse amplitude in normal subjects (n=7) was 19.2 mL/(min 100 g) (SD5.0), which was higher than the average value of 9.5 mL/(min 100 g) in the patients with PAD. These results indicate a high diagnostic ability of the pulse amplitude measured in the toe for diagnosing PAD.

AUC: area under the curve

## Discussion

In the present study, we examined the potential of the toe blood flow and pulse amplitude measured with a portable LDF as a screening test for PAD in HD patients compared with the diagnostic abilities of ABI and TBI. The main findings were that the pulse amplitude measured with the portable LDF correlated well with the ABI and TBI. Its sensitivity and specificity for detecting PAD were also rather high.

The ABI is used as a general reference test for PAD screening. However, due to the effects of arteriosclerosis, the high possibility of false-negative results in HD patients has been pointed out. The sensitivity of ABI to detect PAD is reported to be low (29.9%) in HD patients.^3)^ In the present study, the specificity of ABI for diagnosing PAD using a cutoff value of ABI<0.9 was 100%, and the sensitivity was 83%. This sensitivity is insufficient for a reliable diagnosis of PAD, but it is higher than that reported from a previous study,^3)^ probably due to the low rate of arteriosclerosis in the patients included in this research. The TBI provides a measure of the degree of ischemia in the lower limbs below the ankle joint and is useful for the early detection of PAD.^10)^ In the present study also, the sensitivity at a cutoff value of TBI of <0.6 was 100% (although the specificity was only 75%), indicating that the TBI showed a sufficient diagnostic ability for PAD in the dialysis patients included in the present research, consistent with previous reports.^10)^

The portable LDF was developed to evaluate the functions of the autonomic nervous system by measuring the blood flow at the skin surface. While portable LDF has been applied in managing several diseases,^11–13)^ its usefulness has not yet been evaluated for diagnosing PAD in dialysis patients. Since the pulse amplitude measured with the portable LDF showed a high diagnostic value for PAD in dialysis patients and the measurement could easily be made during an HD session, we consider that measurement of the pulse amplitude with a portable LDF is a superior to measurement of the ABI and TBI as a screening tool for PAD. Toe blood flow and changes in the toe blood flow have been reported as useful diagnostic indicators of PAD in dialysis patients.^8,9)^ However, in the present study, we found that the sensitivity of the toe pulse amplitude for the detection of PAD was higher than that of the toe blood flow; that is, the toe pulse amplitude measured with the portable LDF showed sufficient sensitivity for the diagnosis of PAD in dialysis patients. In diagnosing PAD, when the values of ABI/TBI are below the reference values, angiography is performed to confirm the diagnosis of PAD. Although there are numerous reports on ABI and TBI, and reference values have been established, measurements of these parameters are not suitable as screening tests for the diagnosis of PAD in dialysis patients, as the measurements cannot be conveniently made during dialysis sessions, and the preparation time for the measurements are also longer. On the other hand, a portable LDF is very easy to use, and the toe pulse amplitude can be measured during dialysis sessions and is well-correlated with ABI and TBI. Therefore, measuring the pulse amplitude with the portable LDF is considered more suitable as a screening test for PAD in patients undergoing dialysis.

The precise reason for the high sensitivity of the pulse amplitude measured with the portable LDF for detecting PAD remains unclear. A possible explanation is that the pulse amplitude may reflect stenosis or occlusion of the blood vessels in the leg. Since an LDF measures the blood flow at a shallow depth from the skin’s surface, the measured flow rate with the LDF could be affected by the heterogeneity in skin perfusion,^14)^ psychological stress, and underlying diseases such as diabetes mellitus. This could explain the poor detection ability of the measured blood flow in the toe for PAD. On the other hand, the measured pulse amplitude in the toe is the difference between the maximum and minimum values of the blood flow rate, so it may reflect the ease of transmission of the blood flow waveform throughout the lower extremities; this could explain the low pulse amplitude in patients with PAD.

This study had a few limitations. First, the study included only a small number of patients. Ishii et al. used LDF to measure the blood flow rates in the dorsal and plantar surfaces of the feet in dialysis patients. They enrolled 128 (including 22 patients on PAD) and showed that the blood flow on the plantar surface was significantly lower in patients with PAD (sensitivity 0.88 and specificity 0.78).^9)^ In this study, using LDF, we performed an exploratory study to determine whether measurement of the blood flow rate and pulse amplitude with an LDF might be an effective screening tool for PAD if the toe is selected as the measurement site, and found that the pulse amplitude measured in the toe was a rather suitable screening tool for PAD. Therefore, multicenter clinical trials with large sample sizes will be needed to confirm the diagnostic ability of the pulse amplitude measured in the toe for PAD in dialysis patients. Second, the sensitivity of ABI was relatively high in the patients included in the present study, indicating that the patients included in this study did not have severe atherosclerosis. Further study is required to confirm if it might be possible to detect PAD from the pulse amplitude measured in the toe in patients in whom ABI is judged as showing a false-negative result by measuring the pulse amplitude with the degree of vascular stenosis or occlusion determined by diagnostic imaging tests, such as angiography, CT angiography, magnetic resonance imaging angiography, and ultrasonography.

## Conclusion

The pulse amplitude in the toe measured with a portable LDF showed a potentially high sensitivity for diagnosing PAD in HD patients. The measurement with the portable LDF can be easily performed even during a dialysis session, with no need for any specialized knowledge or skill. LDF may be a powerful tool for use in screening tests for PAD.
